# Droplet Transportation on Janus Harp Wires for Enhanced Fog Harvesting

**DOI:** 10.1002/smll.202506765

**Published:** 2025-07-31

**Authors:** Yutaka Yamada, Taku Ishikawa, Kazuma Isobe, Akihiko Horibe

**Affiliations:** ^1^ Faculty of Environmental, Life, Natural Science and Technology Okayama University Okayama 700‐8530 Japan; ^2^ Graduate School of Environmental, Life, Natural Science and Technology Okayama University Okayama 700‐8530 Japan

**Keywords:** droplet transport, fog harvesting, janus wire, wettability difference

## Abstract

Ensuring freshwater resources is a vital issue for human beings worldwide. Fog harvesting is one promising way to provide water from unconventional sources. However, clogging by the captured liquid depresses the fog harvesting performance. Here, a harp‐shaped Janus harvesting system, which has thin wires with a superhydrophobic side facing the fog stream and a superhydrophilic back side to transport the droplets, is used to yield simultaneous fog capturing and water transport abilities. Attached droplets on the Janus wire transported along the periphery avoided clogging and enhanced the performance. The Janus system thus suppressed the increase and fluctuations of actual shade coefficients, which indicated blockage of the fog stream. This optimized the design of the harvester. Experiments using a multilayered Janus harvester demonstrated a significant enhancement compared with that constructed with mono‐wettability wires. Overall, the results indicated the promise of droplet transportation on single wires for improving fog harvesting, as well as for other applications such as oil mist recovery and demulsification.

## Graphical abstract

Janus thin wires, with superhydrophobic wettability on one side and superhydrophilic wettability on the opposite side, are used for enhanced fog water harvesting. Droplet motion along the circumferential direction helped to avoid clogging while maintaining larger shade coefficients that enhance the harvesting performance. Up to five stacked layers of wire arrays are evaluated.

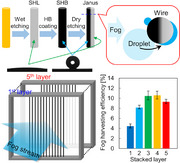

## 1 Introduction

Freshwater, which is a critical resource for human life, is facing scarcity.^[^ ^1^, ^2^, ^3^ ^]^ Population growth, socio‐economic development, and changing consumption patterns will further increase water demands.^[^ ^4^ ^]^ To overcome this issue, especially in arid regions, tiny water droplets floating in fog have been a potential source of freshwater.^[^ ^5^, ^6^, ^7^ ^]^ Therefore, droplet harvesting has been investigated in terms of mechanisms, techniques, and materials.^[^ ^8^, ^9^, ^10^, ^11^, ^12^, ^13^, ^14^ ^]^

The mechanism of fog harvesting can be simply divided into two parts.^[^ ^15^ ^]^ The first is a capturing process involving collisions of fog droplets on a solid harvester or on a liquid attached to the harvester. The second process is liquid transportation, whereby the captured liquid is transported to a reservoir for utilization or to prevent a drain through evaporation and detachment. The capturing process is governed by aerodynamic characteristics of the harvester and deposition properties,^[^ ^16^ ^]^ while the transportation depends on liquid mobility on the harvester. To maximize water harvesting, many types of fog harvesters have been investigated; simple mesh and harp‐shaped structures have been especially attractive.^[^ ^17^, ^18^, ^19^, ^20^ ^]^ However, they suffer from clogging, which disrupts liquid transportation and blocks the fog stream.^[^ ^10^, ^17^ ^]^

A simple way to avoid clogging is to increase the spacing between adjacent solid materials, but this decreases the harvesting performance. Thus, transporting droplets before clogging occurs is a critical issue. Therefore, wettability and shape gradients inspired by those in animals and plants^[^ ^21^, ^22^, ^23^, ^24^, ^25^, ^26^ ^]^ have been investigated for droplet transportation. In particular, wettability differences along a longitudinal direction of the wire can be controlled via the surface structure and chemical composition, resulting in successful droplet transportation.^[^ ^27^, ^28^, ^29^, ^30^ ^]^ The shape gradient typically seen as a conical wire found on cactus spines^[^ ^22^, ^23^ ^]^ generates a Laplace pressure difference between a tip (thin) side and root (thick) side in attached droplets that drives droplets to the root side. This mechanism is also seen on spindle‐knot structured fibers inspired by spider silk.^[^ ^24^, ^25^ ^]^ The droplet motion driven by these mechanisms helps to collect attached droplets on the harvester and increases the amount of harvesting.^[^ ^28^, ^30^ ^]^ However, these mechanisms remove other attached droplets on the wire along the transport direction. Although this reduces clogging, the decrease in area to collect the fog droplets reduces the harvesting performance. To overcome this trade‐off, droplet motion along the fog flow direction was investigated by modifying the wettability of the harvester.

To achieve this, a Janus system, which has a hydrophobic feature on one side and a hydrophilic feature on the opposite side, was applied on mesh, membrane, and foam materials.^[^ ^31^, ^32^, ^33^, ^34^, ^35^, ^36^, ^37^, ^38^ ^]^ The fog harvesting performance was enhanced with the more hydrophobic side facing the fog stream because captured droplets on this side were transported to the rear hydrophilic side before detachment. On the other hand, the opposite side reduced the performance due to the absence of the droplet transportation.^[^ ^31^ ^]^ However, even though the Janus system could perform directional liquid transportation through the system, drainage from the more hydrophilic side relied on gravitational forces acting on the liquid. Therefore, liquid remaining on the hydrophilic side disturbs the fog stream through the harvester and suppresses performance. The reason why liquid remained was due to stronger pinning force acting on the receding contact line^[^ ^39^ ^]^ compared to the gravitational force; thus, a reduced contact line would be helpful for fast drainage. In addition, once the clogging occurs at the mesh or other materials, it is difficult to recover to the unclogged state. Accordingly, the harp‐type structure is promising because the absence of horizontal lines decreases the contact line length. From this point of view, a staggered wire array which has superhydrophobic and superhydrophilic wires at upstream and downstream side was investigated.^[^ ^40^, ^41^ ^]^ Successful droplets transpotation between wires were observed, and it enhanced the fog harvesting performance. However, it was sensitive to an interval of both wires. Furthermore, superhydrophilic wires had to be used despite their poor fog harvesting ability.

In the present work, we fabricated superhydrophobic (SHB)/superhydrophilic (SHL) Janus wires to achieve droplet transportation on one wire. Furthermore, a harp‐shaped harvester was constructed to demonstrate the effects of droplet transportation on fog harvesting performance. Droplets captured at the superhydrophobic region facing the fog stream were transported along the circumferential direction of the wire and drained at the superhydrophilic region at the rear. This behavior suppressed fluctuations at the fog collection area and enhanced the harvesting performance.

## 2 Results and Discussion

### 2.1 Sample Characterization

**Figure 1:**
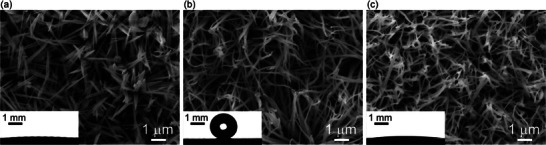
Scanning electron microscopy images of a) superhydrophilic, b) superhydrophobic, and c) Janus surfaces. Inset panels show images for contact angle measurements.

**Figure** **1** shows scanning electron microscopy images of wires. Needle‐like structures with diameters less than 1 µm were fabricated via chemical etching, as shown in Figure 1a. A static contact angle *θ* was estimated to be 3° ± 1° from an image of a deposited droplet, which indicated a highly wettable SHL surface. Figure 1b shows similar surface structures after a hydrophobic coating had been applied. In this case, the contact angle was 156° ± 3°, with advancing and receding contact angles (*θ*_a_ and *θ*_r_) from the SHB surface of 158 ± 3° and 152 ± 2°, respectively. The contact angle hysteresis ∆*θ* = *θ*_a_ – *θ*_r_ was less than 10° because of the limited solid‐liquid contact area. Figure 1c shows a structure after treatment with dry etching on one side of the Janus surface. It was similar to that for the SHB wire surface, but had a contact angle of 2° ± 1°, which was comparable to that for the SHL surface. This indicated successful removal of the hydrophobic coating via etching. However, the contact angle at a masked (not etched) region was 154°, which indicated the SHB state. Thus, a significantly large wettability contrast was achieved on the Janus wire surface.

### 2.2 Droplet Removal Behavior

**Figure 2:**
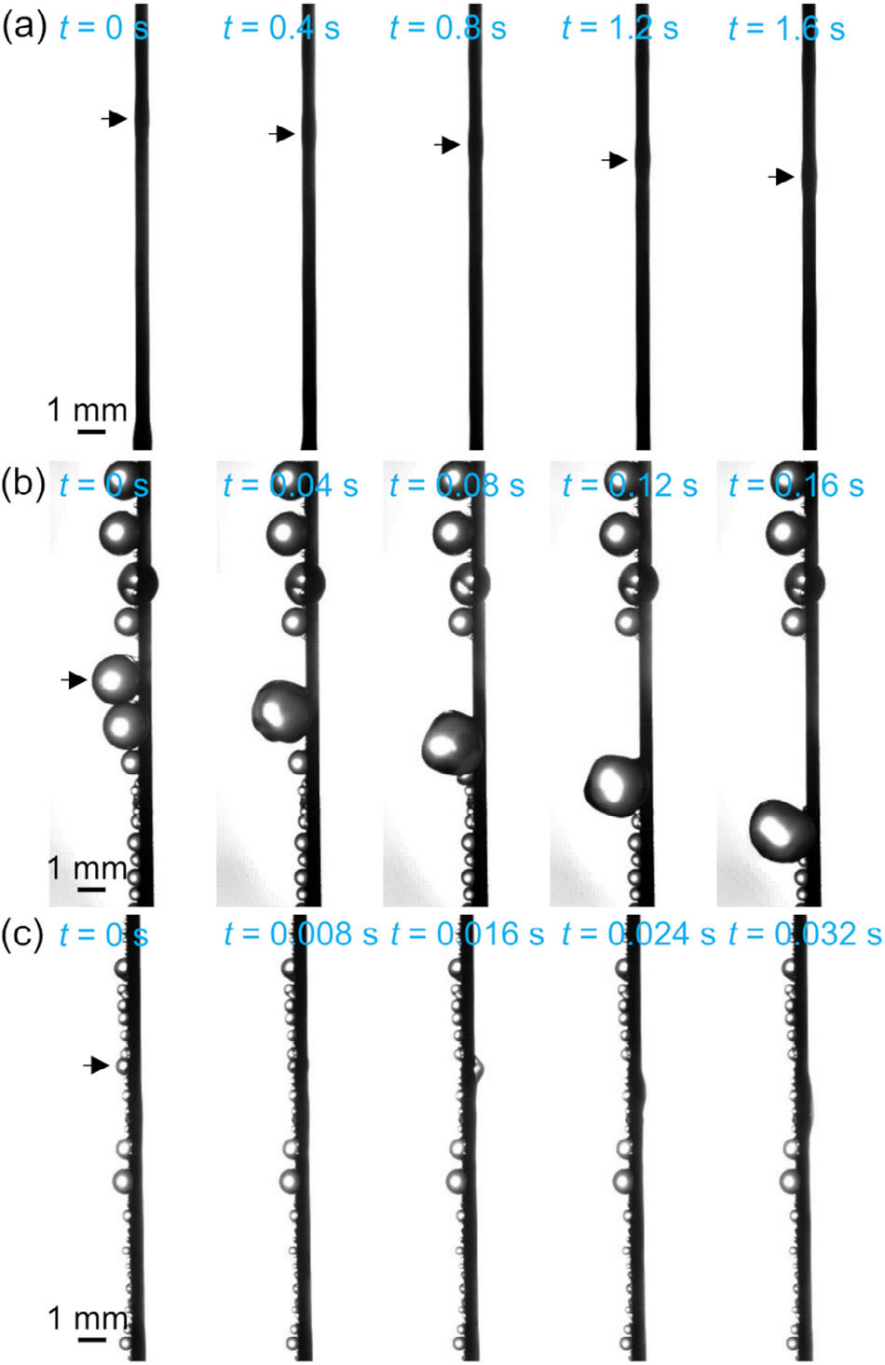
Droplet removal behavior on a) superhydrophilic, b) superhydrophobic, and c) Janus wires. The fog velocity was set at 1.5 m s^−1^.

The effect of wire wettability on droplet behavior during fog harvesting was evaluated. Details of the behavior are revealed in **Figure** **2** (see Movie S1–S3, Supporting Information, S1), which shows typical liquid removal from each wire. The fog stream was directed from left to right. A liquid film was formed on the SHL wire (Figure 2a) and an oval‐shaped droplet, indicated by arrows, flowed downward. In Figure 2b, droplets grown on the SHB wire rolled‐off and swept away other droplets attached below, as reported previously.^[^ ^40^, ^41^ ^]^ This resulted in the re‐exposure of the droplet‐free surface while the fog capturing area was reduced. For the Janus wire (Figure 2c), the droplets grew on the upstream side and were transported to the circumferential direction of the wire as shown by the arrow, and then flowed down the wire. This motion thus indicated that the Janus feature effectively transported droplets from the upstream to the downstream sides. Droplets attached below the transported droplets remained in their locations and helped to capture further fog droplets by expanding the area facing the fog stream.

**Figure 3:**
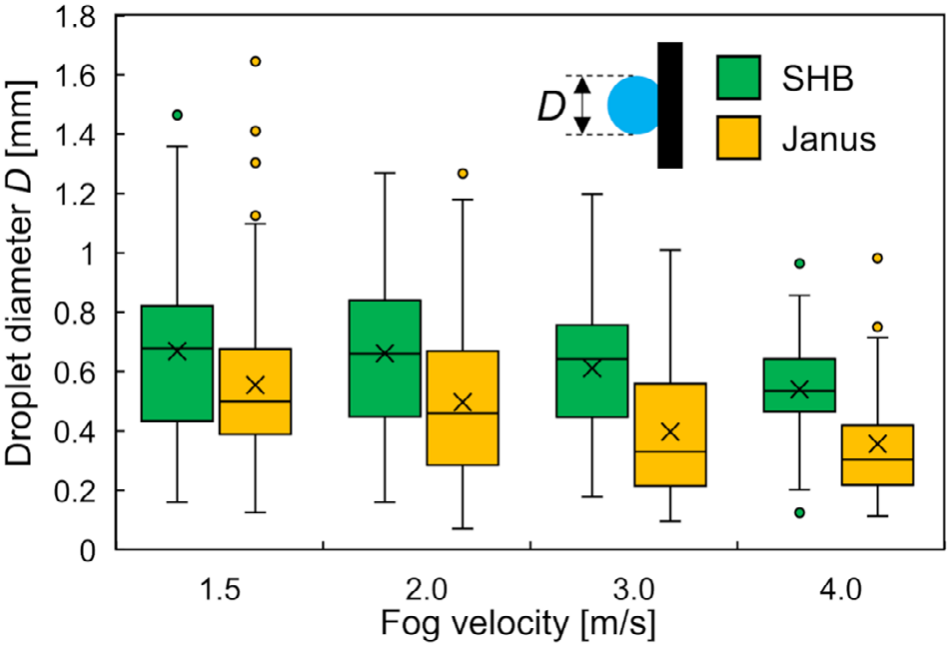
Distributions of removed droplets from superhydrophobic and Janus wires.

The sizes of transported droplets are summarized as box plots in **Figure** **3**. The droplet diameter *D* just before transportation or shedding was used as the characteristic length, and 100 droplets for each condition were analyzed to establish the size distributions. Although the results had a wide range, there was a trend in which smaller droplets attached to the Janus wires were transported relative to those on SHB wires. Here, the wettability on the upstream sides of both wires was SHB; therefore, droplets were captured and grown on the surfaces. During this process, the contact line of the droplet shifted to the side of the wire because of the fog flow and droplet growth. Then, the three‐phase contact line approached the SHL region located at the rear side. Hence, the droplets on the Janus wire were transported in a circumferential direction. This behavior occurred before droplet removal that was dominant for SHB wires. In addition, for both wires, the diameter *D* decreased with increasing fog velocity. This was because the fluctuating droplet motion that initiated transportation was enhanced by increasing the fog velocity.

### 2.3 Fog Harvesting Performance at One Layer of the Harvester Array

**Figure 4:**
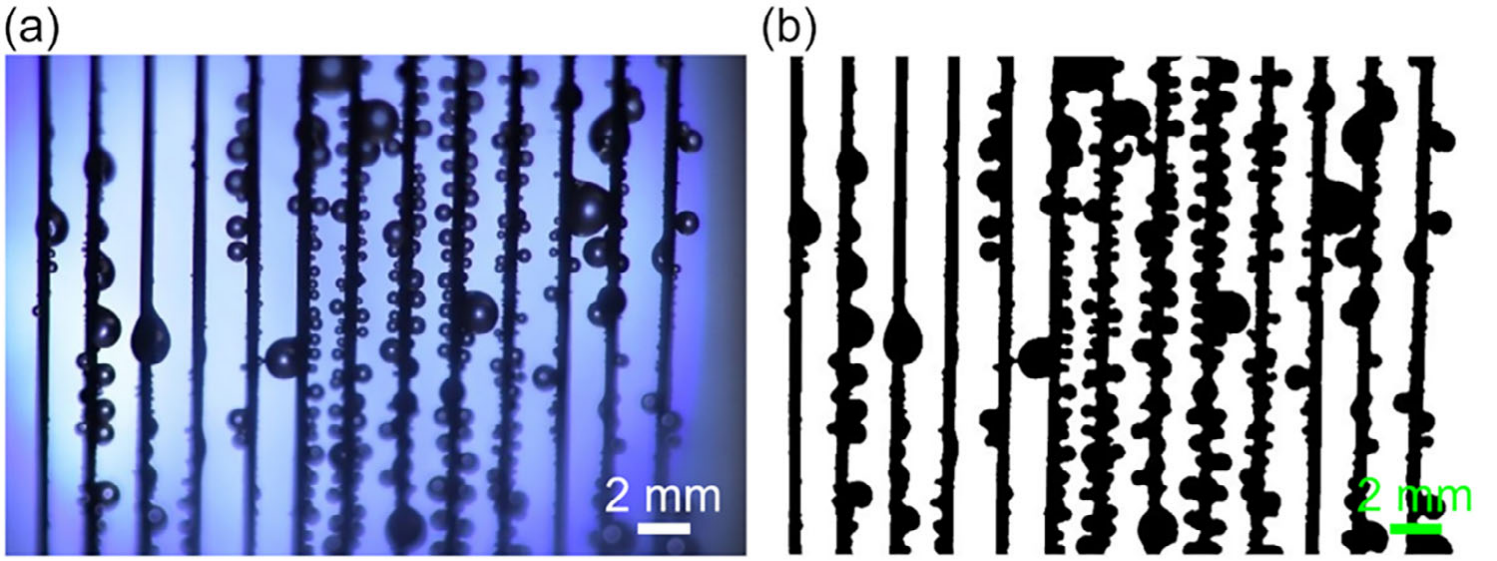
a) Snapshot during the experiment and b) binary processed image of the same snapshot for actual shade coefficient analysis.

To investigate the effect of droplet removal behavior on the fog harvesting performance, we first conducted experiments using one layer of the harvester array. **Figure** **4**a shows a typical image of the harvester during the experiments. The masses of droplets collected by the harvester and attached droplets were measured as the amount of harvested water $\dot{m}$. In addition, the performance was evaluated as the overall efficiency η_exp_, given by:

(1) $$\eta_{\exp}=\frac{\dot{m}}{Q}$$

where *Q* is the mass flow rate of water in the fog stream. However, the harvesting efficiency η depends on the aerodynamic, capture, and drainage efficiencies η_ac_, η_cap_, and η_dr_, respectively, and can be estimated by:^[^ ^15^, ^16^ ^]^

(2) $$\eta =\eta_{ac}\eta_{cap}\eta_{dr}$$

The aerodynamic efficiency η_ac_ indicates the fraction of upwind fog flow that passes through the harvester, which depends on the pressure drop caused by the harvester. A previous report showed that this relation was given by:^[^ ^16^ ^]^

(3) $$\eta_{ac}=\frac{SC_{geo}}{1+\sqrt{C_{0}/C_{d}}}$$

where *C*_0_ is the pressure drop coefficient for the harvester and *C*_d_ is the drag coefficient for an impermeable plate. Here, *C*_0_ is a function of the geometrical shade coefficient *SC*_geo_, and is given by:

(4) $$C_{0}=K_{Re}\left[1.3SC_{geo}+{\left(\frac{SC_{geo}}{1-SC_{geo}}\right)}^{2}\right]$$

where *K*_Re_ is an empirical correction factor that depends on the flow characteristics.

The capture coefficient η_cap_ indicates whether fog droplets follow or deviate from the air stream; i.e., deviated droplets will collide with the harvester and be captured. This is described by the Stokes number *St* given by:

(5) $$St=\frac{2\rho_{water}v{r_{fog}}^{2}}{9\mu r_{wire}}$$

where ρ_water_, μ, and *r*_wire_ are the water density, air viscosity, and wire radius, respectively. *r*_fog_ is the radius of the fog droplets, which was 10 µm in the experiments (See Supporting Information, S2). Using *St*, the capture coefficient can be expressed as:^[^ ^42^ ^]^

(6) $$\eta_{cap}=\frac{St}{St+\pi /2}$$

The drainage efficiency represents the loss of captured liquid and is mainly governed by re‐entrainment by the stream and evaporation.^[^ ^15^ ^]^ The former refers to detachment of the droplets from the harvester, which was observed at increased fog velocities. However, detached droplets were much smaller than the rolled‐off and transported droplets. Therefore, we neglected it here. Evaporation was driven by the difference in vapor pressure at the liquid surface and that in foggy air. Although non‐saturated air was introduced in the fog generation chamber, the humidity should have been increased until it was blown by the outlet. Therefore, evaporation was neglected here and η_dr_ = 1 was assumed.

Although the harvesting efficiency could be estimated by using Equations ((2), (3), (4), (5), (6)), attached droplets on the harvester increased the blockage fraction of the fog stream, as shown in Figure 4a. Hence, the actual shade coefficients (*SC*_a_) were analyzed experimentally. Captured movies were binarized as shown in Figure 4b, and then counted as black areas. The cross‐sectional area of the fog stream was used to divide the actual blocking area, and hence *SC*_a_ could be estimated.

**Figure 5:**
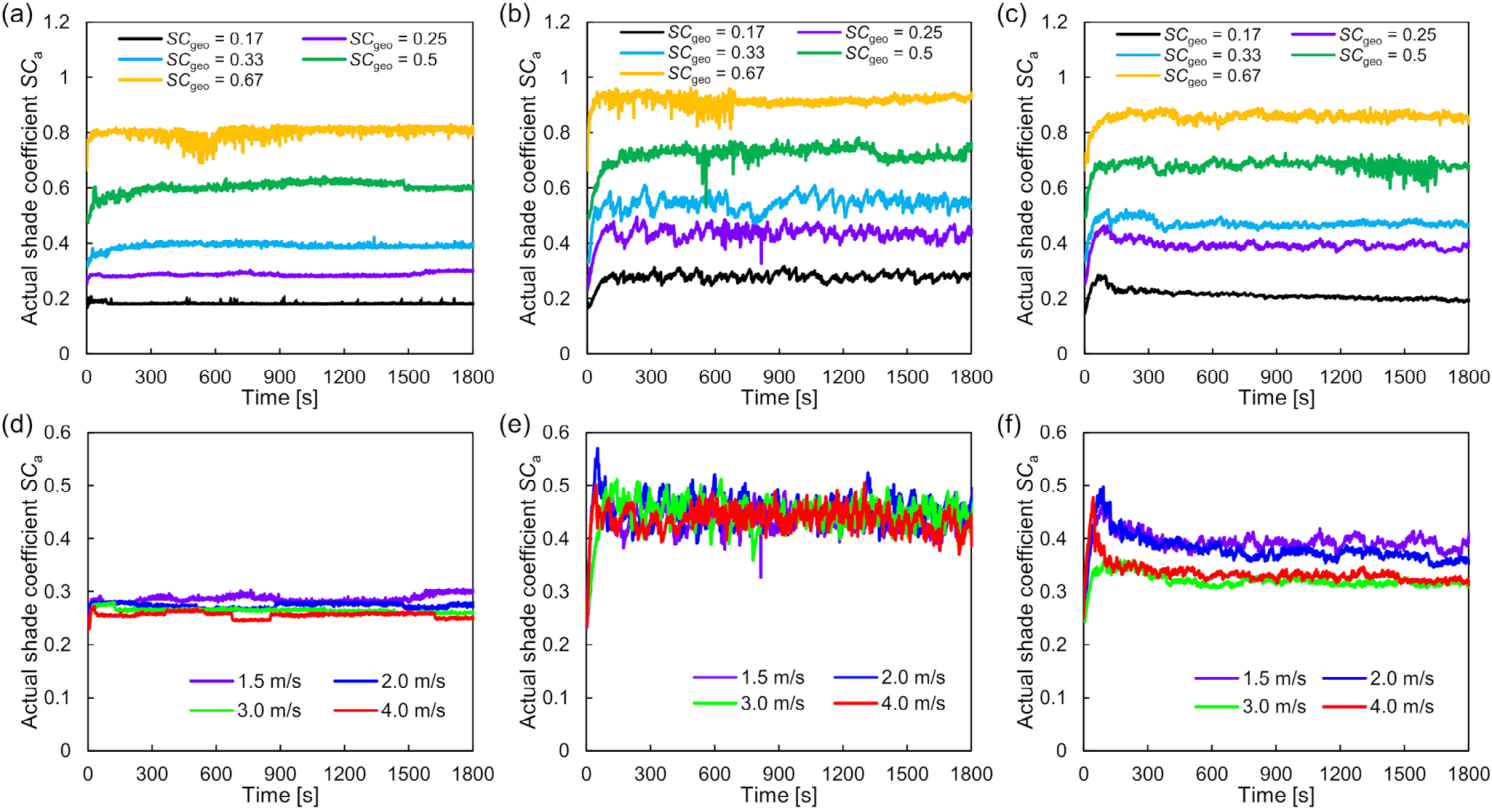
Actual shade coefficient *SC*_a_ as a function of time. a–c) show the relation at each geometrical shade coefficient (*SC*_geo_) obtained at 1.5 m s^−1^. d–f) show the relation at each fog velocity at *SC*_geo_ = 0.25. (a) and (d), (b) and (e), and (c) and (f) show the results at superhydrophilic, superhydrophobic, and Janus wettability, respectively.

**Figure** **5** shows the transition of *SC*_a_ as a function of time. It increased at the beginning of the experiments because of the collection of fog droplets and then reached a quasi‐steady state at 100–300 s. Figure 5a–c shows analyzed *SC*_a_ at 1.5 m s^−1^ for each *SC*_geo_ at SHL, SHB, and Janus wire arrays, respectively. Owing to the formation of liquid films on the wires, the increase in *SC*_a_ was small for the SHL wire array. The increasing ratio was larger for SHB and Janus wire arrays relative to that for the SHL array because droplets with large contact angles enlarged the blocking area. However, the results for the Janus wire array were slightly smaller than those for the SHB array because of droplet transportation. In addition, a *SC*_a_ fluctuation shows different trends. A small fluctuation on the SHL wire array was caused by downflow of oval‐shaped droplets shown in Figure 2a. The sweeping effect of droplet removal on the SHB wire, shown in Figure 2b, was reflected as an intermittent drop in *SC*_a_ in Figure 5b. However, droplet transportation on the Janus wires kept other droplets on the wires; therefore, the Janus *SC*_a_ fluctuation was smaller than that of SHB. Figure 5d–f shows *SC*_a_ values depending on the fog velocity at *SC*_geo_ = 0.25. While the SHL and SHB wire arrays exhibited no significant differences, *SC*_a_ decreased with increased fog velocity for the Janus wire array, as shown in Figure 5f. The increased fog velocity decreased the size of transported droplets on the Janus wires, as shown in Figure 3, and, as a result, larger droplets that increase *SC*_a_ could not remain on the wires.

**Figure 6:**
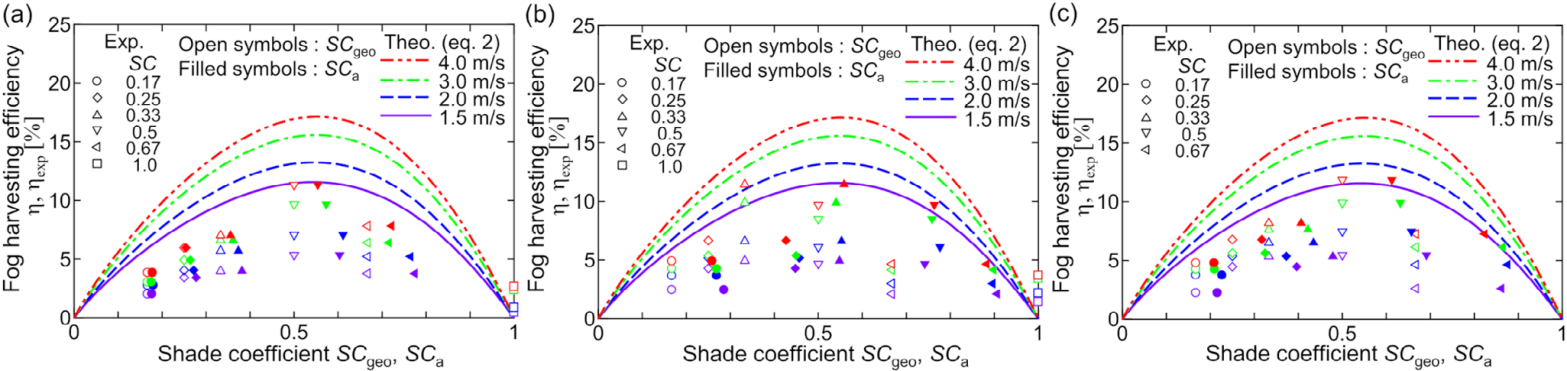
The fog harvesting efficiency depending on the geometrical shade coefficient (*SC*_geo_) and the fog velocity at a) superhydrophilic, b) superhydrophobic, and c) Janus wire arrays. Open plots show the *SC*_geo_ while filled plots show the actual shade coefficient (*SC*_a_). Lines show the theoretical efficiency estimated via Equation 2. Colors of plots indicate the fog velocity that follows the line color.

**Figure** **6** shows the fog harvesting efficiency as a function of the shade coefficient and fog velocity. Open and filled symbols indicate *SC*_geo_ and *SC*_a_, respectively. Here, impermeable plates with SHL and SHB wettabilities were used to obtain the data at *SC*_geo_ = 1.0, and are shown in Figure 6a,b. The general trend was that increased fog velocity increased efficiency because of the increased η_cap_. In the relationship between efficiency and *SC*, the efficiency had a maximum with increased *SC* and then decreased. Furthermore, the lowest efficiency was for the impermeable plates. These results follow theoretical estimations as indicated by lines in Figure 6 and previous experimental results^[^ ^31^, ^43^, ^44^ ^]^ whereas experimental results were lower than theoretical values. One of this reason could be the estimation of the Stokes number. As previously reported by Lee et al.,^[^ ^31^ ^]^ the velocity of fog flow decelerated near the liquid attached on the harvester. But this local velocity can not be considered in the current discussion because of the difficulty of the averaged velocity measurement and the dependence on the droplet shape. Therefore, further discussion based on the detailed velocity measurement is needed. Regarding the relationship between the *SC* and the efficiency, *SC*_geo_ = 0.5 exhibited the highest efficiency for SHL and Janus arrays (Figure 6a,c), whereas that for SHB occurred at *SC*_geo_ = 0.33. This did not correspond to the theory. However, plots represented by *SC*_a_ agreed well with the trends indicated by theoretical estimations. Hence, using the actual shade coefficients *SC*_a_ were better when comparing results with theoretical estimations.^[^ ^44^ ^]^ In addition, maximum efficiency values obtained via experiments were η_exp_ = 5.46%, 7.47%, 9.92%, and 11.87% for fog velocities *v* = 1.5, 2.0, 3.0, and 4.0 m s^−1^, respectively, and these were obtained at the Janus wire array. These results indicate the effectiveness of droplet transportation to improve fog harvesting efficiency.

### 2.4 Fog Harvesting Performance with Multilayers

**Figure 7:**
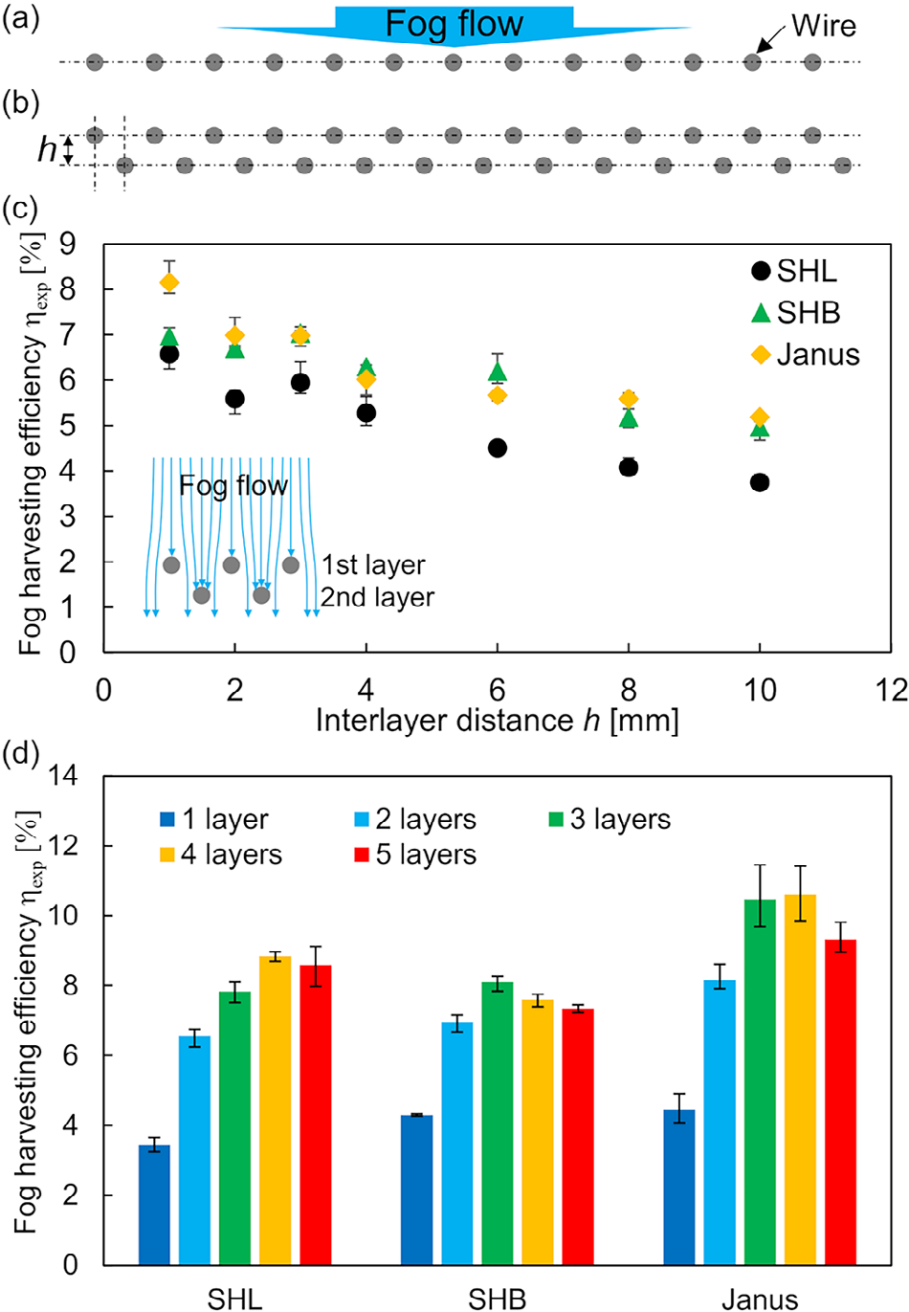
Schematic of the fog harvesting wire array at a) 1 layer and b) multilayers. Geometrical shade coefficient (*SC*_geo_) of each layer is 0.25, and these are separated by the interlayer distance *h*. Multilayered harvester is arranged staggered location. c) Fog harvesting efficiency as a function of the interlayer distance *h*. Fog velocity and layer number of the harvester are 1.5 m s^−1^ and 2 layers. The inset illustration shows the schematic of the fog droplets trajectory incoming to the two‐layered harvester. d) Relation between fog harvesting efficiency and the number of stacked wire array layers at 1.5 m s^−1^.

An increase in total efficiency for fog harvesting is vital to confronting water scarcity. Therefore, a multilayered harvester is discussed below. *SC*_a_ = 0.5–0.6 exhibited the highest performance for single‐layered harvesters, as shown in Figure 6. Further increases in *SC*_a_ are expected by stacking harvesters along the fog stream, which will diminish the performance. Hence, *SC*_geo_ = 0.25 was used for the arrangement of a single layer, as shown in **Figure** **7**a. While a staggered and aligned arrangements are choices for multilayered harvester (See Supporting Information, S3), a second layer was located in the staggered position at an interlayer distance *h* (Figure 7b) ranging over 1–10 mm. Here, droplets grown on the latter layer can be transported to the front layer at the Janus wire array, but a liquid bridge will be formed at the SHB wire array. However, these behaviors were not observed at *h* larger than 2 mm because droplets were transported or rolled‐off before reaching other wires.

Figure 7c shows the fog harvesting efficiency at *v* = 1.5 m s^−1^ as a function of *h*. The efficiency showed the maximum value at *h* = 1 mm of the Janus wire array while it decreased with increased *h*, and similar trend was observed for other wettabilities. In addition, *h* = 0 mm corresponded to the single‐layer arrangement with *SC*_geo_ = 0.5, where η_exp_ = 5.36%, 4.67%, and 5.46% for SHL, SHB, and Janus, respectively. These indicate greater than 20% enhancement by introducing the interlayer distance *h* = 1 mm. This can be caused that fog droplets circumvented by the first layer passed through the interspace and were then collected at the second layer (See Supporting Information, S4, Movies S4–S8, Supporting Information). This effect was diluted by increasing the interlayer distance *h*, and was no longer effective at *h* ≈8 mm. Figure 7d shows the harvesting efficiency at *v* = 1.5 m s^−1^ as a function of the number of stacked layers, where *h* = 1 mm. As previously reported,^[^ ^15^, ^19^, ^45^ ^]^ the harvesting efficiency increased with increased stacking number. Our results showed same trend and η_exp_ = 10.60% was obtained for four layers of Janus wire arrays as a maximum value. The efficiency then decreased with further increases in layers for all wettabilities. In particular, the SHB wire array reached an efficiency maximum value at three layers; it was less than the other two wires because of fog stream blockage. As shown in Figure 5, the SHB array exhibited a larger *SC*_a_ than the other two arrays by attaching droplets, but no droplet transportation. *SC*_a_ will increase further when stacking layers (See Supporting Information, S5), reducing fog flow through the wire arrays. Hence, the harvesting efficiency of the SHB wire array was promptly curtailed by further stacking, whereas that for the Janus wire array was enhanced.

To discuss the fog harvesting capability, a rough estimation is considered on the basis of the obtained results. The maximum harvesting efficiency in the multilayered experiment (η_exp_ = 10.60%) corresponded to 20.35 kg m^−2^ 30 min^−1^, where the harvester area facing the fog stream was assumed to be 25 mm × 25 mm. **Table** **1** shows fog harvesting rates using Janus materials.^[^ ^31^, ^32^, ^33^, ^34^, ^35^, ^36^, ^37^, ^38^ ^]^ Although parameters such as the fog velocity and flow rate are different, and types of harvesters is different, our result showed relatively high harvesting rate. From the practical viewpoint, the World Health Organization has reported that 20 L person^−1^ day^−1^ is likely to be sufficient for drinking, cooking, food hygiene, and hand/face washing.^[^ ^46^ ^]^ This is not sufficient for other hygiene practices. To ensure all food and personal hygiene, including bathing and laundry at the home, more than 100 L person^−1^ day^−1^ is recommended.^[^ ^46^ ^]^ On the basis of these suggestions, our results seem to be adequate for personal water use. However, the amount of water in the fog flow in our experiments was estimated to be more than a factor of 100 denser than actual fog.^[^ ^47^, ^48^ ^]^ Accordingly, practical fog harvester should be designed to refer the harvesting efficiency instead of the estimated yield from the experimental condition. In addition, the fog velocities and directions with respect to the harvester may not be consistent. Therefore, further enhancement of the harvesting performance is necessary by considering the actual *SC* by varying the fog velocity. The results here indicate that effective droplet transportation along the circumferential direction of a vertically installed wire will help to optimize harvesters.

**Table 1:** Comparison of the experimental conditions and fog harvesting performance using Janus mesh, harp, and other materials.

Type	Material	Wire Diameter	*SC*_geo_	Fog Velocity	Fog Flow Rate	Harvesting Rate	Temperature, Relative Humidity	Refs.
Mesh	PLA	1 mm	0.31^a^	1.3 m s^−1^	−	1.3 g cm^−2^ h^−1^	20 ± 2 °C, 80 ± 5%	31
Mesh	Brass	250 ± 13 µm	0.37 ± 0.02	1.86 ± 0.25 m s^−1^	0.22 ± 0.04 L h^−1^	4.7 kg m^−2^ h^−1^	22.8 ± 0.5 °C, 87 ± 4%	32
Foam	Copper	−	−	0.5 m s^−1^	−	3.7 g cm^−2^ h^−1^	Room temperature, no RH data	33
Mesh	Copper	40 µm	0.72^a)^	0.5 m s^−1^	0.07 g s^−1^	2.2 g cm^−2^ h^−1^	18 °C, 90%	34
Membrane	PET	−	−	1 m s^−1^	−	5.5 g h^−1^ at 30 spines	No temperature data, 95%	35
Mesh & absorbent	Copper & cotton	150 µm	0.61^a^	0.7 m s^−1^	−	0.31 ± 0.03 g 5 min^−1^ at 2 × 2 cm^2^	−	36
Membrane	PAN & PDVF	−	−	−	2.5 mL min^−1^	88.5 ± 6.0 mg cm^−2^ min^−1^	20 °C, 84%	37
Membrane	Copper & PVDF‐HEP	150 mesh	−	0.7 m s^−1^	−	1821.51 ± 56.18 mg cm^−2^ h^−1^	−	38
harp	Copper	0.5 mm	0.5	1.5 m s^−1^	240 ± 10 g h^−1^	20.35 kg m^−2^ 30 min^−1^	20.5 ± 1.0 °C, 52 ± 10%	This work

^a)^ Geometric shade coefficient was calculated by dimensions of pore and wire.

## 3 Conclusion

Fabricated wires with Janus dual wettability enhanced fog harvesting performance because of SHB and SHL regions. Single‐wire experiments showed effective droplet transportation along the circumferential direction of Janus wires, while droplets attached to SHB wires were often shed or blocked fog flow. The fog harvesting performances of single‐layer wire arrays were evaluated by varying the geometrical shade coefficient *SC*_geo_ of the harvesters and the fog velocity. The harvesting efficiency first increased with increased *SC*_geo_ and then decreased, regardless of wire wettability; this trend followed theoretical estimations. Although *SC*_geo_ values at the maximum efficiency depended on the wettability, the wire array with an actual shade coefficient *SC*_a_ ranging from 0.5 to 0.6 was optimized to maximize the efficiency. This result indicated the importance of *SC*_a_ for design optimization. Multilayered harvesters were also evaluated, and the effect of the interlayer distance *h* indicated that *h* = 1 mm exhibited the highest performance. Increasing the number of harvester layers increased the amount of captured water; however, more than five layers depressed the performance. Furthermore, while the droplet removal process on each wire showed less difference on the performance in the single‐layer harvesters, significant improvements were obtained for the multilayered harvesters. This was because there was less blockage area for the fog stream in the Janus wire array due to the occurrence of droplets transportation. The multilayer maximum efficiency (η_exp_ = 10.60%) corresponded to 20.35 kg m^−2^ 30 min^−1^ in heavy fog conditions.

## 4 Experimental Section

### Wire Preparation

Three types of wires were prepared. Details were reported elsewhere.^[^ ^40^, ^41^ ^]^ Briefly, copper wires with a diameter of 0.5 and 65 mm lengths were used as the base material. They were cleaned in ethanol (Fujifilm Wako Pure Chemical Co., Japan) via ultrasonication for 15 min and rinsed in purified water (CPW‐102, Advanted Toyo Ltd., Japan). They were then immersed in 6 M aqueous hydrochloric acid (Fujifilm Wako Pure Chemical Co., Japan) for 30 s to remove the oxide layer and then rinsed. The surface structures of the wires were fabricated via a wet process. They were immersed in a mixed aqueous solution of sodium hydroxide (2.5 M) (Fujifilm Wako Pure Chemical Co., Japan) and ammonium persulfate (0.1 M) (Fujifilm Wako Pure Chemical Co., Japan) for 20 min at 4 °C and then rinsed. After drying with compressed air, they were baked for 2 h at 180 °C to dehydrate Cu(OH)_2_ at the surfaces. The process up to this point was for the preparation of superhydrophilic (SHL) wires. Superhydrophobic (SHB) wires were prepared to modify the surface wettability of SHL wires. In this case, Teflon AF 1600X (Chemours Co., US) was used as the modifier and was dissolved in FC‐770 (Hayashi Pure Chemical Ind. Ltd., Japan) at a concentration of 0.5 wt.%. Wires were dipped into the solution for 30 s, and then dried and heated at 90 °C for 60 min. Wires with Janus wettability were prepared as follows. SHB wires were placed on a jig plate that had 0.5 mm grooves (both width and depth). The jig plate was then placed in an etching chamber (RIE‐10N1, Samco Co., Japan) to remove the outer hydrophobic coating at the upper surface of the wires to expose the underlying SHL surface. The etching was performed at 15 W under 2.0 × 10^−3^ Pa of Ar for a duration of 30 s. In addition, the same types of wettability surfaces were prepared using 15 mm × 15 mm copper substrates for contact‐angle measurements. The etching process was also conducted for the wettability characterization.

### Characterization

Surface structures on each wire were characterized using a scanning electron microscope (JEOL7001F, JEOL Co., Japan). The static contact angle *θ* for each surface was measured via the sessile droplet method. Specifically, 3.5 µL of purified water droplets was gently deposited on each substrate, and the droplet shape was captured with a 100 fps high‐speed camera at a horizontal viewpoint (Cyclone‐1HS, Optronis GmbH, Germany). Static contact angles were analyzed from these images and in‐house software. Advancing and receding contact angles (*θ*_a_ and *θ*_r_) were measured from the behavior of the three‐phase contact line during injection and extraction of the droplet volume.

### Fog Harvesting Experiments

**Figure 8:**
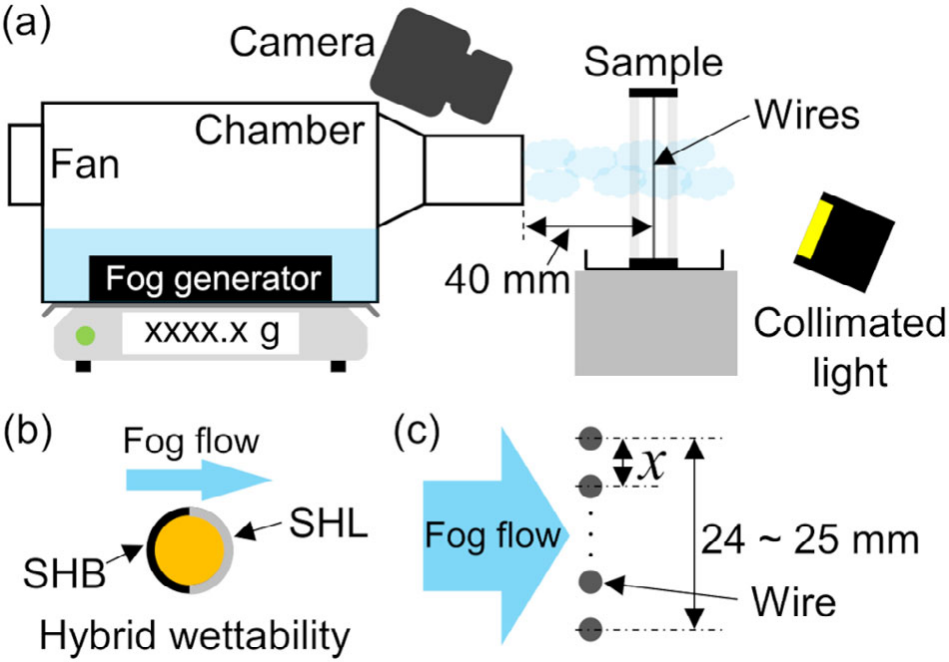
a) Schematic of the experimental setup for fog harvesting. b) Top view of the Janus wire placement. c) Top view of the wire array arrangement at single layer.

A schematic of the experimental setup is shown in **Figure** **8**a. Fog generation chamber took in an ambient air and fog generated by an ultrasonic fog generator (IM4‐36D/S, Seiko Giken Inc., Japan) was flowed from an outlet that had a cross‐sectional area of 25 mm × 25 mm. Fog generation rate in the chamber was 240 ± 10 g h^−1^, which makes more than 100 times denser fog compared with actual fog.^[^ ^47^, ^48^ ^]^ However, aerodynamic and capture coefficients are independent on the fog concentration. Therefore, the harvesting efficiency can be evaluated with the current setup as long as evaporation of captured droplets can be negligible. The fog stream velocity *v* was measured by an anemometer (6006‐D0, Kanomax Japan Inc., Japan) before each experiment and was varied over the range of 1.5–4.0 m s^−1^ by changing the voltage applied to the fan installed in the chamber. Hence, the fog concentration decreased with the increase of the velocity. A sample consisting of wires mounted in an acrylic frame with opening area of 70 mm × 62 mm was placed 40 mm from the fog outlet. The SHB regions of the Janus wires faced the fog stream, as shown in Figure 8b. Temperature (20.5 ± 1.0 °C) and relative humidity (52 ± 10%) of an ambient air were maintained for all experiments, and corresponding absolute humidity was 9.3 ± 2.4 g.

The droplet removal behavior from the wires during fog harvesting was captured with the high‐speed camera from a horizontal direction (Figure 8a). Because droplet shedding from the SHB wire and droplet transfer from the SHB region to the SHL region on the Janus wire were both rapid, they were recorded at 1000 fps. By contrast, flowing liquid films on the SHL wire were captured at 100 fps.

The fog harvesting performances of the harp‐shaped harvesters were characterized for various wire arrays. The arrays were constructed with wires having the same wettability and were arranged perpendicular to the fog flow, as shown in Figure 8c. The spacings (*x*) between the wires were 0.75, 1.0 1.5, 2.0, and 3.0 mm, with a total array width (*w*) of 24–25 mm. These corresponded to geometrical shade coefficients *SC*_geo_ = 0.67, 0.5, 0.33, 0.25, and 0.17, respectively, which were defined as the blockage fraction facing normal to the fog stream. However, collisions of fog droplets on the wires that make liquid films on the SHL surfaces or large droplets on the SHB surfaces will change the fog blockage areas. Therefore, an actual shade coefficient *SC*_a_ was analyzed using a movie recorded with a digital camera (D5300, Nikon, Japan). In addition, the wire array shown in Figure 8c was defined as one layer; up to five layers were stacked along the fog stream direction to characterize multilayer harvesting performance. Performances were measured at least three times for each condition, and the duration of each run was 30 min.

## Conflict of Interest

The authors declare no conflict of interest.

## Supporting information



Supporting Information

Supplemental Movie 1

Supplemental Movie 2

Supplemental Movie 3

Supplemental Movie 4

Supplemental Movie 5

Supplemental Movie 6

Supplemental Movie 7

Supplemental Movie 8

## Data Availability

The data that support the findings of this study are available from the corresponding author upon reasonable request.
