# Supplementary material for: Droplet Transportation on Janus Harp Wires for Enhanced Fog Harvesting
Source: Small. 2025 Jul 31;21(37):e06765. doi: 10.1002/smll.202506765 (PMC12444825; doi:10.1002/smll.202506765)
Supplement: Supplementary file 1 — Supporting Information [file SMLL-21-e06765-s002.docx]

Supporting Information

Droplet Transportation on Janus Harp Wires for Enhanced Fog Harvesting

*Yutaka Yamada^*^, Taku Ishikawa, Kazuma Isobe, Akihiko Horibe*

**S1. Movies of droplet removal process on each wire**

Movie S1 shows droplet removal process from the superhydrophilic (SHL) wire. Fog flow was directed from left to right. Played back at 1/5× speed.

Movie S2 shows droplet removal process from the superhydrophobic (SHB) wire. Fog flow was directed from left to right. Played back at 1/50× speed.

Movie S3 shows droplet removal process from the Janus wire. Fog flow was directed from left to right. SHB part is facing to the upstream side. Played back at 1/50× speed.

**S2. Droplet size distribution**

A glass slide which coated by thin layer of silicone oil was used to capture fog droplets and the size of these droplets were measured. Figure S1(a) shows typical image of captured droplets visualized by an optical microscope (VW-9000, Keyence Co., Japan). The size of more than 150 droplets were measured by these images and the size distribution was obtained as shown in Figure S1(b). The radius scattered in wide range while the average radius was obtained as 10 μm.


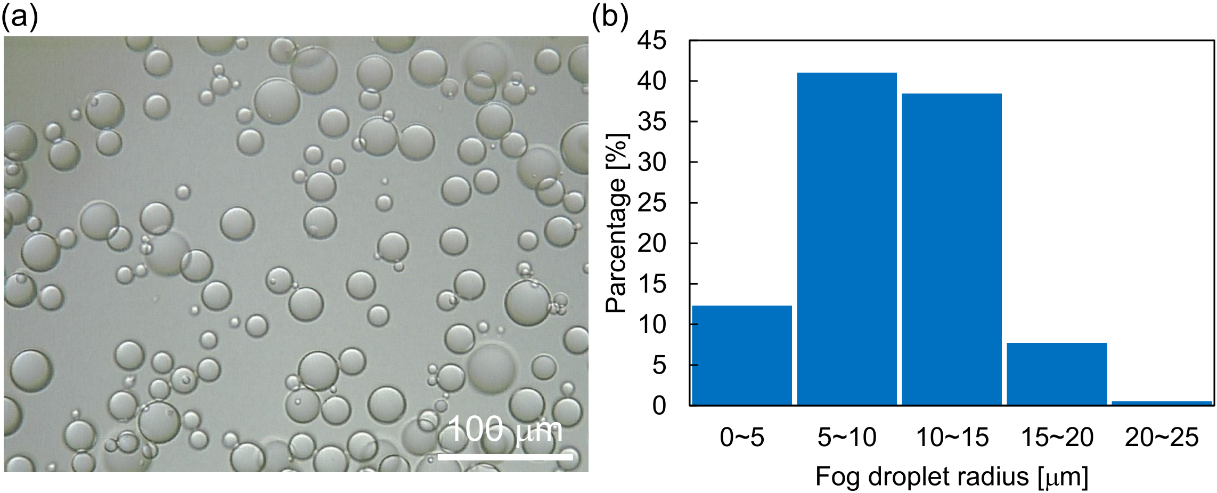


Figure S1 (a) Optical microscope image of fog droplets on silicone oil. (b) Size distribution of fog droplets.

**S3. Arrangement of multilayered harvester**

To determine the optimal multilayered harvester setup, an arrangement and interlayer distance *h* were varied. Here, the staggered and aligned arrangement shown in Figure S2 was prepared and *h* was varied from 1 to 10 mm. Fog velocity was set at 1.5 m/s.

Figure S3 shows the result of the fog harvesting efficiency with each wire wettability. Regardless of the wettability, *h* = 1 mm with staggered arrangement showed the highest efficiency and then the efficiency decreased as the increase of the interlayer distance *h*. On the other hand, the efficiency at the aligned arrangement showed minimum value at *h* = 1 mm because fog flow was blocked by the first layer. The efficiency showed maximum values at *h* = 2 ~ 4 mm and then decreased as the increase of *h*. Though the efficiency of both arrangements showed comparable at *h* > 8 mm, that of the staggered arrangement showed higher value compared with the aligned arrangement regardless of *h*.


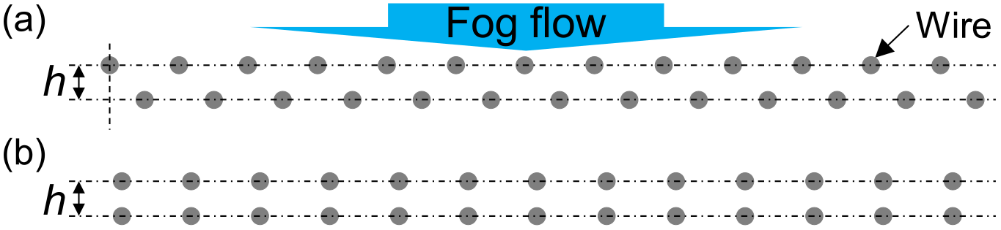


Figure S2 Type of multilayered harvester arrangements. (a) Staggered and (b) aligned arrangements.


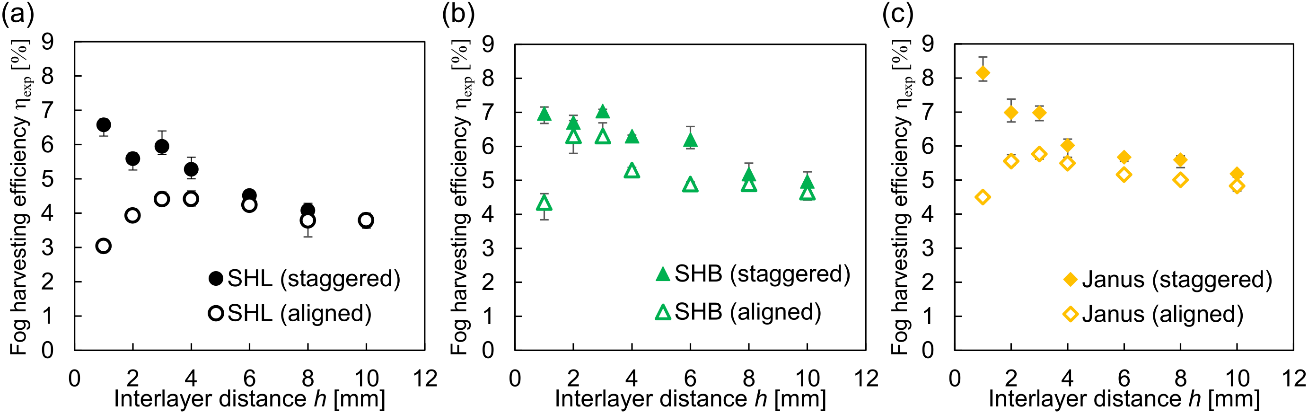


Figure S3 The fog harvesting efficiency as a function of the interlayer distance *h* and layer arrangements. (a) SHL, (b) SHB, and (c) Janus wire arrays.

**S4. Flow field visualization**

Fog flow around the harvester was visualized by the experimental setup shown in Figure S4(a). Vertically installed wire was placed 40 mm from the fog outlet. Laser sheet (MSDL532-16010131, M SQUARE Co., Japan) was used to irradiate the central cross section (*xy* plane) of the fog stream. Figure S4(b) shows the schematic image of the *xy* plane of fog stream. Laser sheet was irradiated from the bottom of the observation area. For the visualization experiment, the fog velocity was adjusted 1.5 m/s. Due to the droplets attached on the wires disturb the observation, SHL wires are used. High-speed camera (Cyclone-1HS, Optronis GmbH, Germany) mounted above the wire was used for the flow visualization and images were recorded at 100 fps with the exposure time of 1000 μs. In addition, the fog flow directed from left to right in whole of visualization.


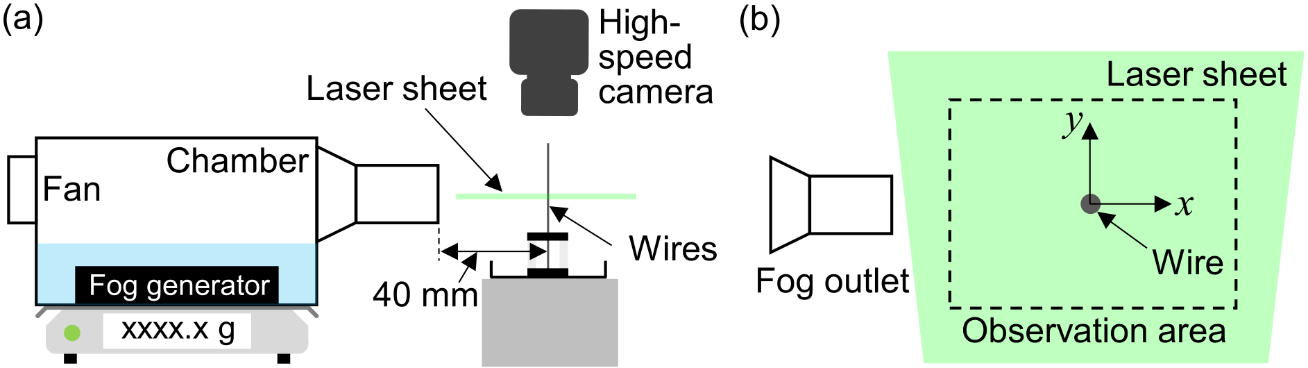


Figure S4 (a) Schematic of the experimental setup for flow field visualization. (b) Top view of the observation area.

Since the high-speed camera captures reflected light by the fog droplets, denser fog will appear as bright image. Therefore, a relation between the fog flow rate and the brightness of captured images is analyzed before the flow field visualization around the wire. Here, an input voltage was varied to change the amount of generated fog, and images were captured for 100 s. Figure S5(a) shows typical snapshot of the fog stream and Figure S5(b) shows an averaged image. Because an artifact was appeared in the edge region of the averaged image, we calculated the averaged brightness by excepting 200 pixels from the edge (i.e. the area enclosed by the blue line was used for calculation.). Obtained relation is shown as Figure S6. Though obtained relation was not linear, we obtained the trend that the averaged brightness increases with the increase of the fog flow rate.


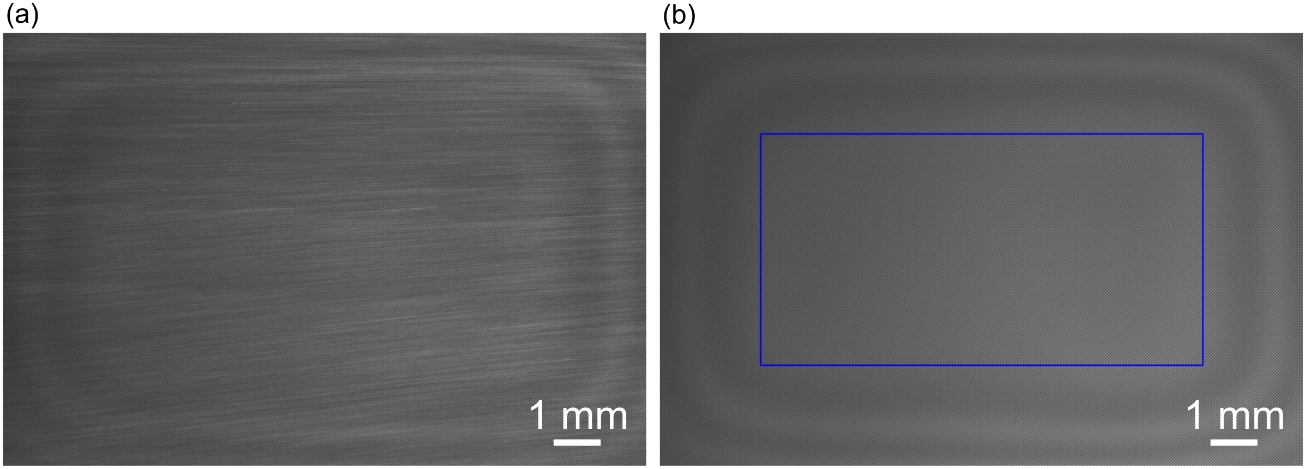


Figure S5 (a) Snapshot of fog flow without the fog harvester. (b) Image averaged from 10000 (= 100 s × 100 fps) snapshots. Due to the artifact, the area in the blue rectangle was used for the calculation of average brightness.


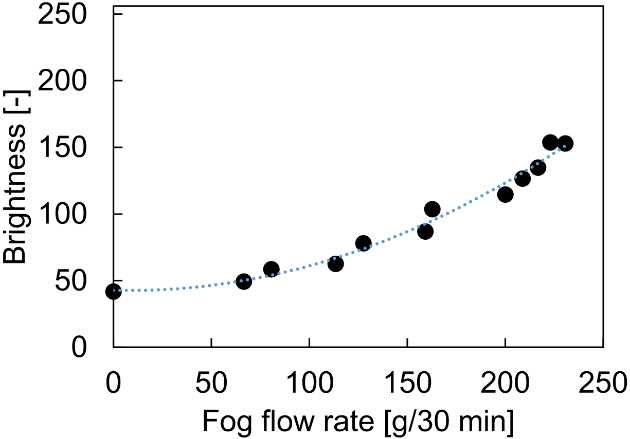


Figure S6 Relation between the fog flow rate and averaged brightness.

Movie S4 shows the flow field around single SHL wire which played back at 1/5× speed. Because the liquid film formed on the wire surface reflects light and it disturbs the visualization, another wire was placed between the light source and the observation area to generate a shade. Vortices generated in the downstream region of the wire were observed clearly. Reynolds number *Re*, which defined as *Re* = *ρvD*/*μ*, where *ρ*, *μ*, and *v* are the density, viscosity, and velocity of foggy air, and *D* is the diameter of the wire, respectively, is estimated as *Re* **≈** 48, hence the observed vortices are in the transition between twin vortices and Karman vortex. Figure S7(a) shows an averaged brightness of the flow field. To discuss quantitatively, the brightness at sections of *x* = -2, -0.5, and 0.5 mm are extracted and shown as Figure S7(b). Although slight decrease of the brightness with the increase of *y* value are observed in all cross sections due to the distance from the light source, in the area far from the wire showed comparable brightness. However, the brightness of the area behind the wire is reduced.


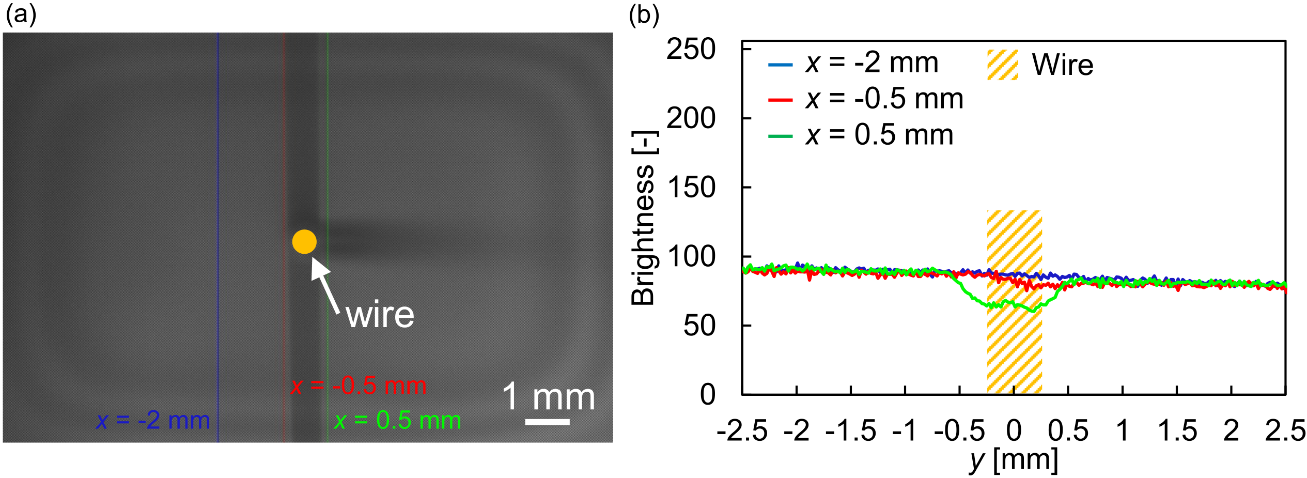


Figure S7 (a) Averaged image of fog flow around the wire. (b) Brightness values at sections of *x* = -2, -0.5, and 0.5 mm from the center of the wire.

Movie S5 shows the flow field around three SHL wires which played back at 1/5× speed. The separation distance between 2 wires at upstream side is 2 mm and the depth from these wires to the wire at downstream side is 1 mm, hence this configuration simulates a part of the multilayered harvester with staggered arrangement (*h* = 1 mm) shown in Figure 7(b) in main manuscript. As seen the movie S5, vortices were generated in the downstream region of the wire located in the second layer instead of wires located in the first layer. Figure S8(a) shows an averaged brightness of the flow field and Figure S8(b) shows brightness profiles at *x* = -3, -0.5, and 0.5 mm, respectively. Due to the presence of the wires at first layer, the brightness was reduced in the area behind these wires. However, the brightness was increased in the area in front of the wire at the second layer. This is because that the fog stream blocked and separated by the wire flowed through the region between wires at first layer. This would help to increase the captured fog droplets and hence the harvesting efficiency was increased by introducing the interlayer distance *h*.


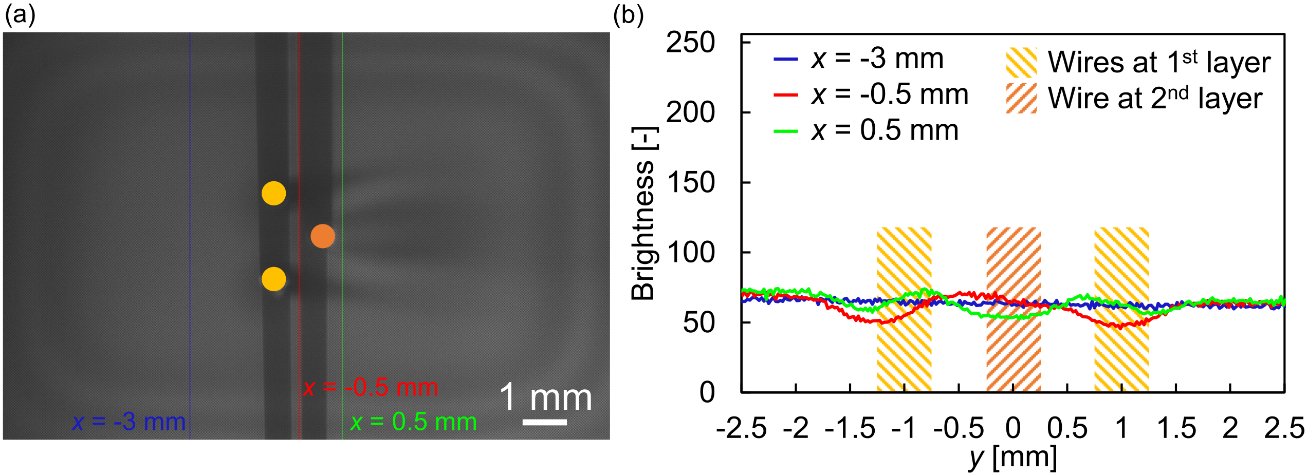


Figure S8 (a) Averaged image of fog flow around a part of the staggered arrangement. An interval between two wires located upstream side is 2 mm, and the interlayer distance *h* is 1 mm. (b) Brightness values at sections of *x* = -3, -0.5, and 0.5 mm from the center of the wire at second layer.

Movie S6 shows the flow field around two SHL wires which played back at 1/5× speed. The interlayer distance *h* is 1 mm, hence this configuration simulates a part of the multilayered harvester with aligned arrangement (*h* = 1 mm) shown in Figure S2(b) in Supporting Information. As shown the movie S6, vortices were seen the downstream region of the wire at second layer. Figure S9(a) shows the average brightness of the flow field and Figure S9(b) shows brightness profiles at *x* = -3, -0.5, and 0.5 mm, respectively. Due to the presence of the wire at first layer, the brightness was reduced and then flowed to the downstream side. Accordingly, the fog harvesting performance at the aligned arrangement was poor compared with that at the staggered arrangement as shown in Figure S3.


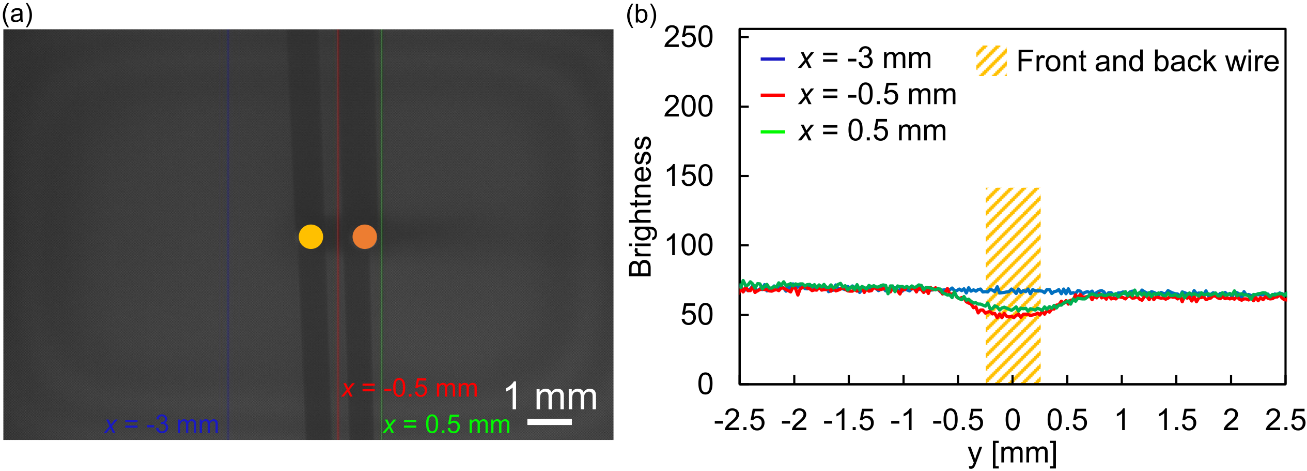


Figure S9 (a) Averaged image of fog flow around a part of the aligned arrangement. The interlayer distance *h* is 1 mm. (b) Brightness values at sections of *x* = -3, -0.5, and 0.5 mm from the center of the wire at second layer.

Movie S7 shows the flow field around the wire array with *SC*_geo_ = 0.5. Movie was recorded at 1000 fps with the exposure time of 998 μs and played back at 1/50× speed. Vortices were generated downstream of each wire.

Movie S8 shows the flow field around the staggered wire array. *SC*_geo_ of each layer is 0.25. Movie was recorded at 1000 fps with the exposure time of 998 μs and played back at 1/50× speed. Vortices were developed downstream of wires at second layer.

**S5. Fog harvesting experiment using multilayered harvester**

Figure S10 shows typical snapshots of the fog harvesting experiment using five layers of wire array. The interlayer distance *h* was set at 1mm. Liquid film was formed on large part of SHL wires surface at 300 s after the start of the experiment shown in Figure S10(a-I). No obvious change was observed over time. For the SHB wire array (Figure S10(b)), droplets grown on wires were observed and liquid bridges formed between neighboring wires or wires at different layer were observed. Though liquid bridges were partially observed on the Janus wire array (Figure S10(c)), the number of droplets attached on wires is significantly smaller than that on the SHB wire array. This fact was caused by the droplet transportation on Janus wires and as a result, the fog harvesting efficiency was enhanced.


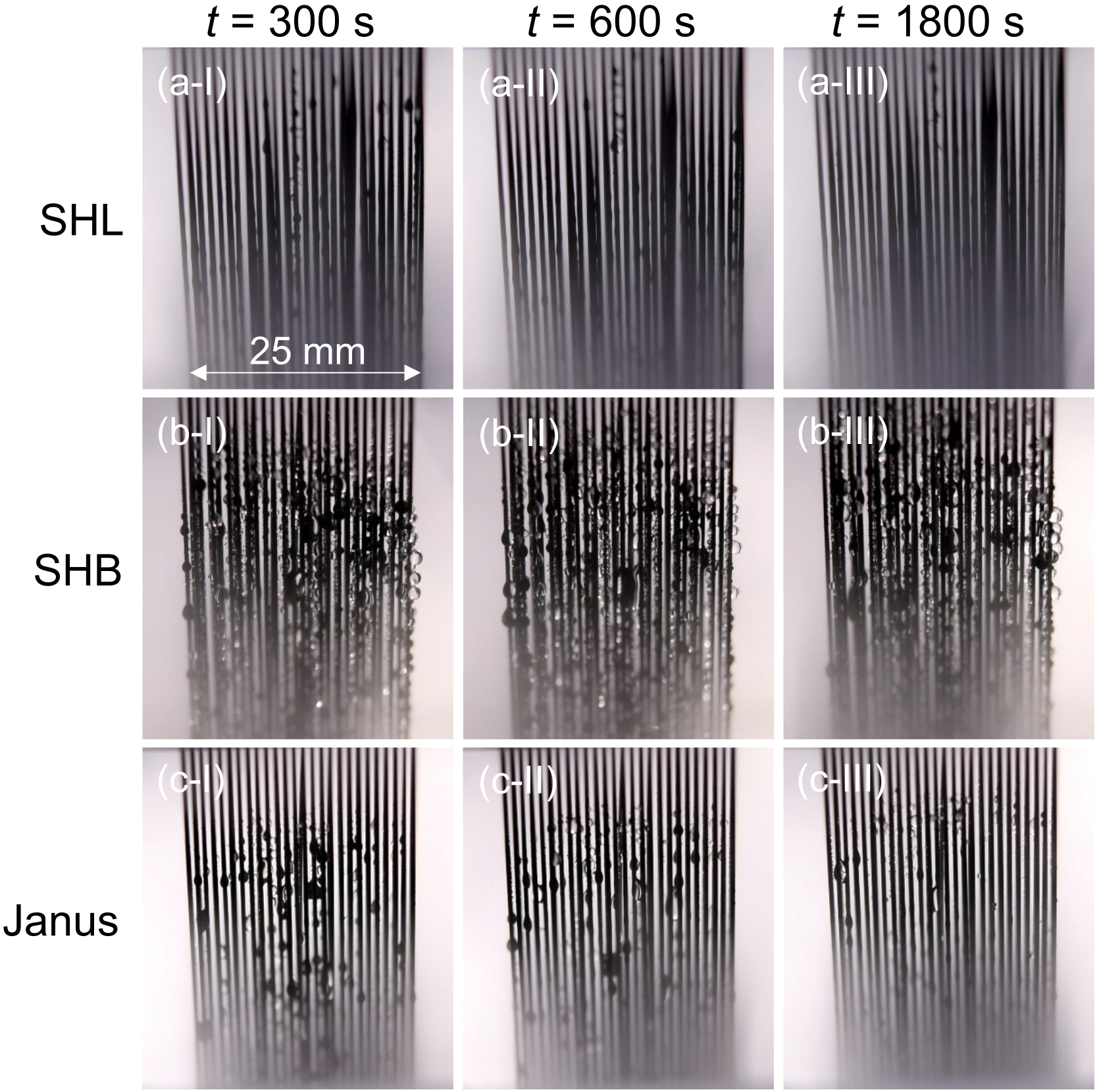


Figure S10 Snapshots of fog harvesting experiments using five layers of (a) SHL, (b) SHB, and (c) Janus wire arrays. 300 s, 600 s, and 1800 s after the start of the experiments are presented.
